# Supplementary material for: Pushing thermal conductivity to its lower limit in crystals with simple structures
Source: Nat Commun. 2024 Apr 8;15:3007. doi: 10.1038/s41467-024-46799-3 (PMC11001610; doi:10.1038/s41467-024-46799-3)
Supplement: Supplementary file 1 — Supplementary Information [file 41467_2024_46799_MOESM1_ESM.pdf]

Supplementary Information: Pushing thermal conductivity to its lower limit in crystals with simple structures

Zezhu Zeng[1,2,†,*], Xingchen Shen[3,†], Ruihuan Cheng[1], Olivier Perez[3], Niuchang Ouyang[1], Zheyong Fan[4], Pierric Lemoine[5], Bernard Raveau[3], Emmanuel Guilmeau[3,*], and Yue Chen[1,*]

[1]Department of Mechanical Engineering, The University of Hong Kong, Pokfulam Road, Hong Kong SAR, China

[2]The Institute of Science and Technology Austria, Am Campus 1, 3400 Klosterneuburg, Austria

[†]These authors contributed equally to this work

[3]CRISMAT, CNRS, Normandie Univ, ENSICAEN, UNICAEN, 14000 Caen, France

[4]College of Physical Science and Technology, Bohai University, Jinzhou 121013, China and

[5]Institut Jean Lamour, UMR 7198 CNRS – Université de Lorraine, 54011 Nancy, France

[*] Correspondence:

zzeng@ist.ac.at;

emmanuel.guilmeau@ensicaen.fr;

yuechen@hku.hk

TABLE S1. Refined crystallographic data of the single-crystal $AgTlI_2$ at 300 K.

Space group	$I4/mcm$ (140)-Tetragonal
λ (Å)	0.71073
Cell	$a(b) = 8.35208(18)$ Å, $c = 7.6628(2)$ Å, $Z = 4$
Volume	534.53(3) Å^3
Reflections to $\theta_{\max}$	36.69°
Rint (%)	3.22
Independant reflections with $I \geq 2\sigma(I)$	434
Refined parameters	11
Reliability factor (%)	1.97

TABLE S2. Refined isotropic and anisotropic atomic displacement parameters (Å^2) of the single-crystal $AgTlI_2$ at 300 K.

Element	U_{iso}	$U_{11} = U_{22}$	U_{33}
Ag	0.0515(7)	0.0613 (10)	0.0319(10)
Tl	0.04128(13)	0.04521(16)	0.0334(2)
I	0.02560(11)	0.02552(13)	0.02577(19)

TABLE S3. Refined crystallographic data at 300 K of $AgTlI_2$ and $AgTl_2I_3$ phases for the powder prepared by the Spark Plasma Sintering (SPS) scheme.

$AgTlI_2$						
Space group	$I4/mcm$ (140)					
Cell	$a(b)$ = 8.3517(1) Å	c =7.6619(1) Å	Z = 4			
Volume	534.42(1) Å^3					
Atomic parameters:						
Atom	Wyckoff position	x	y	z	Occ.	U_{iso}
Ag	4b	0	0.5	0.25	1.000	0.0494
Tl	4a	0.5	0.5	0.75	1.000	0.0332
I	8h	0.18127(3)	0.31873(3)	0.5	1.000	0.0494
$AgTl_2I_3$						
Space group	$R\bar{3}$ (148)					
Cell	$a(b)$ = 10.45(1) Å	c =19.95(1) Å	Z = 9			
Volume	1887.1(1) Å^3					

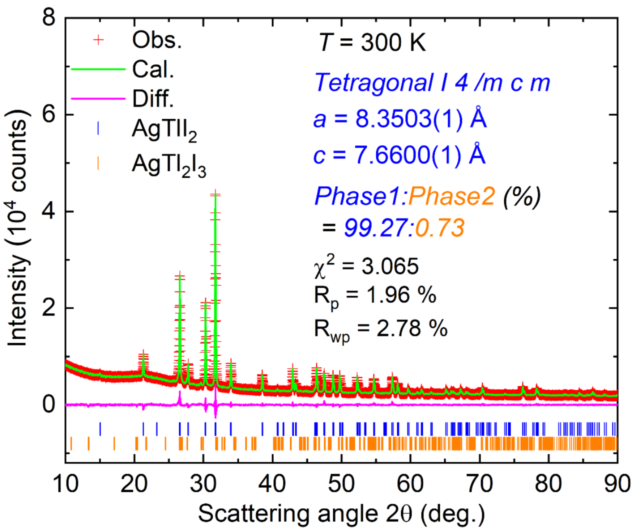

FIG. S1. Rietveld refinements of the as-synthesized powder $AgTlI_2$ XRD data at 300 K.

TABLE S4. Anharmonic scores $\sigma^{\mathrm{A}}_{\mathrm{MD}}$ and the measured thermal conductivities [1–4] for some simple crystals. The data in this table have been utilized to plot Fig. 2b in the main text. We calculated $\sigma^{\mathrm{A}}_{\mathrm{MD}}$ of PbTe, PbSe, AgCl, AgBr, Tl_3VSe_4, TlSe, InTe and $AgTlI_2$ in this work.

Materials	$\sigma^{\mathrm{A}}_{\mathrm{MD}}$	$\kappa^{300\mathrm{K}}_{\mathrm{Exp}}$ (W/mK)	Crystal system	Space group number
Si	0.151	165.6	zincblende	227
InP	0.196	93	zincblende	216
CdTe	0.325	7.5	zincblende	216
CuI	0.41	1.68	zincblende	216
MgO	0.176	60	rocksalt	225
**PbTe**	0.445	2.2	rocksalt	225
**PbSe**	0.303	2	rocksalt	225
NaI	0.36	1.8	rocksalt	225
NaCl	0.315	7.1	rocksalt	225
**AgCl**	0.85	1	rocksalt	225
**AgBr**	0.94	1.1	rocksalt	225
LiI	0.485	1.10	rocksalt	225
CsF	0.478	0.83	rocksalt	225
CdS	0.281	16	wurtzite	186
ZnO	0.243	60	wurtzite	186
GaN	0.151	210	wurtzite	186
$KCaF_3$	0.52	2	Pnma	62
$KCdF_3$	0.535	1.75	Pnma	62
**Tl_3VSe_4**	0.3	0.3	cubic	217
**TlSe**	0.64	0.5	tetragonal	140
**InTe**	0.62	0.7	tetragonal	140
$AgAlS_2$	0.34	1.1	tetragonal	122
$AgAlSe_2$	0.363	0.65	tetragonal	122
$LiGaTe_2$	0.32	0.8	tetragonal	122
$LiInTe_2$	0.345	1.08	tetragonal	122
**$AgTlI_2$**	1.21	0.25	tetragonal	140

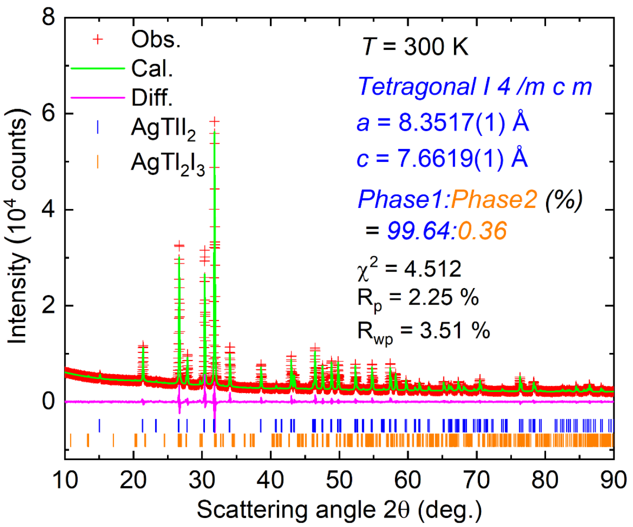

FIG. S2. Rietveld refinements of the SPS-ed powder $AgTlI_2$ XRD data at 300 K.

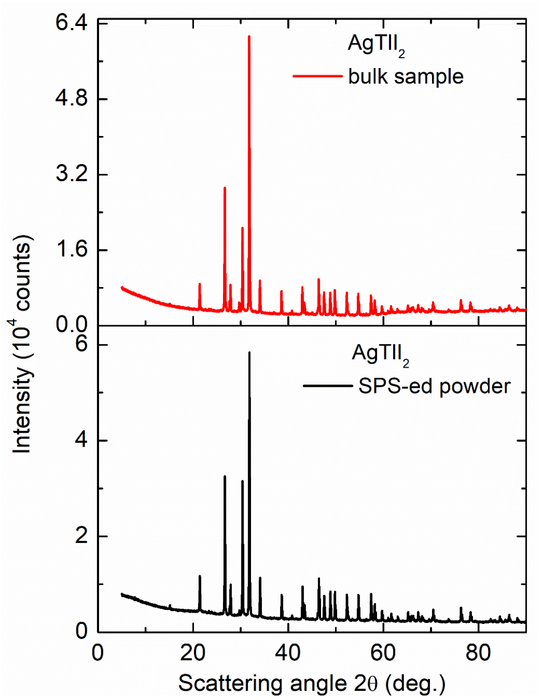

FIG. S3. Top: XRD pattern of the SPS bulk sample (recorded on the surface perpendicular to the SPS pressure direction). Bottom: Powder XRD pattern of the SPS sample.

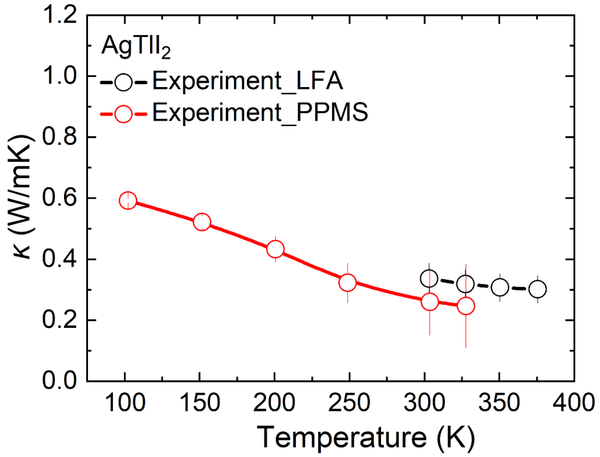

FIG. S4. The experimental thermal conductivities of the SPS-ed bulk $AgTlI_2$ sample measured from LFA and PPMS.

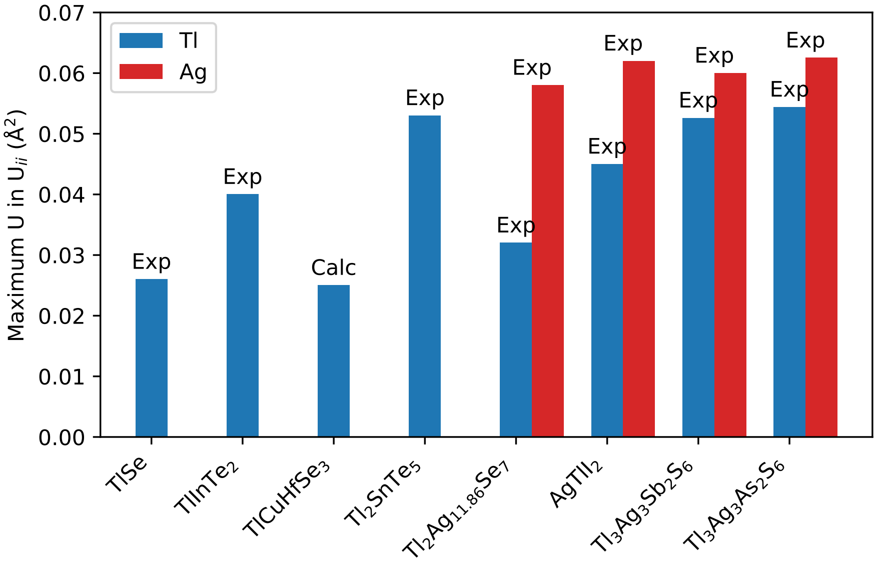

FIG. S5. The maximum anisotropic displacement parameters of Tl and Ag atoms at 300 K in some solid materials with typical rattling behavior. The data for TlSe [5], $TlInTe_2$ [6], $TlCuHfSe_3$ [7], Tl_2SnTe_5 [8], $Tl_2Ag_{11.86}Se_7$ [9], $Tl_3Ag_3Sb_2S_6$ [10] and $Tl_3Ag_3As_2S_6$ [10] was taken from previous experiments and calculations.

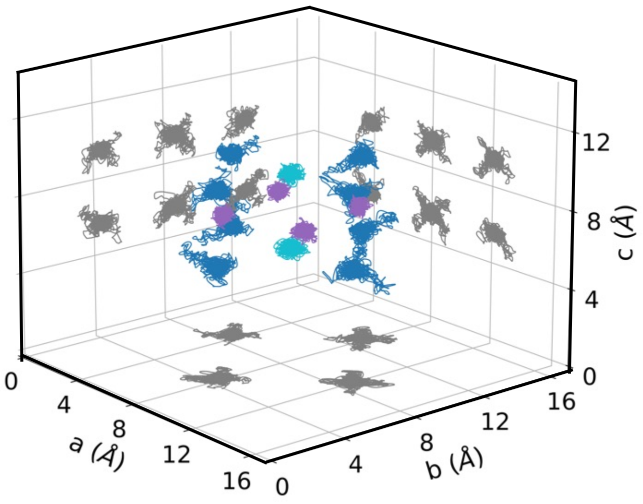

FIG. S6. *Ab initio* molecular dynamics trajectories at 300 K of Ag (blue), Tl (cyan) and I (purple) atoms. The projection of trajectories for six Ag atoms is also shown in the two-dimensional planes.

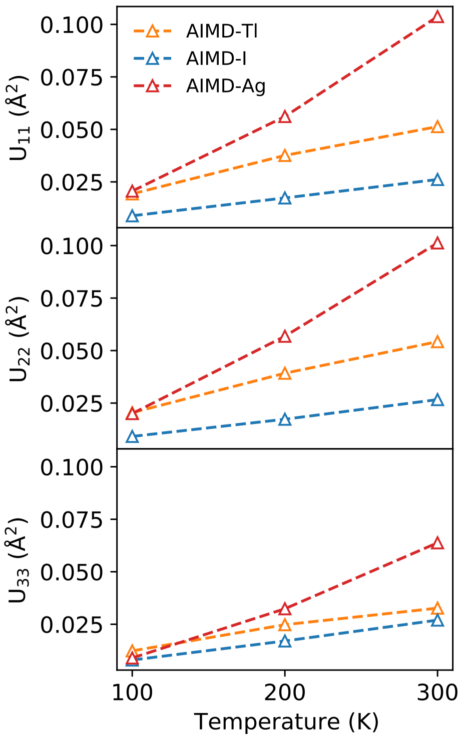

FIG. S7. Anisotropic displacement parameters for Ag, Tl and I atoms from 100 to 300 K calculated from AIMD simulations. Dashed lines are guide for eyes.

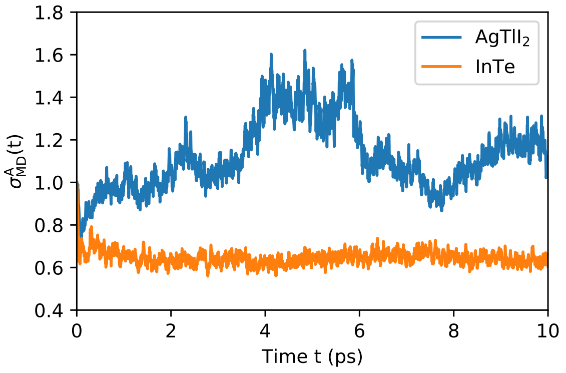

FIG. S8. Time-dependent σ^{A} for InTe and $AgTlI_2$ calculated from AIMD simulations at 300 K.

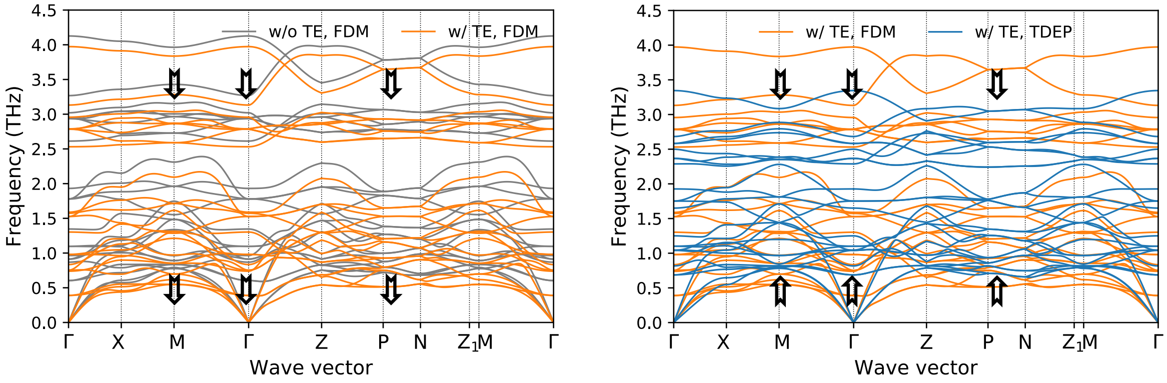

FIG. S9. Phonon dispersions calculated using the finite displacement method (FDM) at 0 K and temperature-dependent effective potential (TDEP) at 300 K. TE represents thermal expansion.

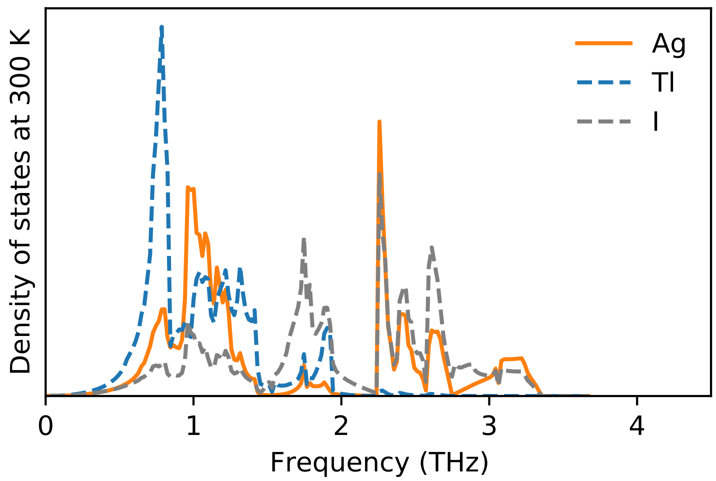

FIG. S10. Projected phonon density of states of $AgTlI_2$ at 300 K calculated using the temperature-dependent harmonic atomic force constants.

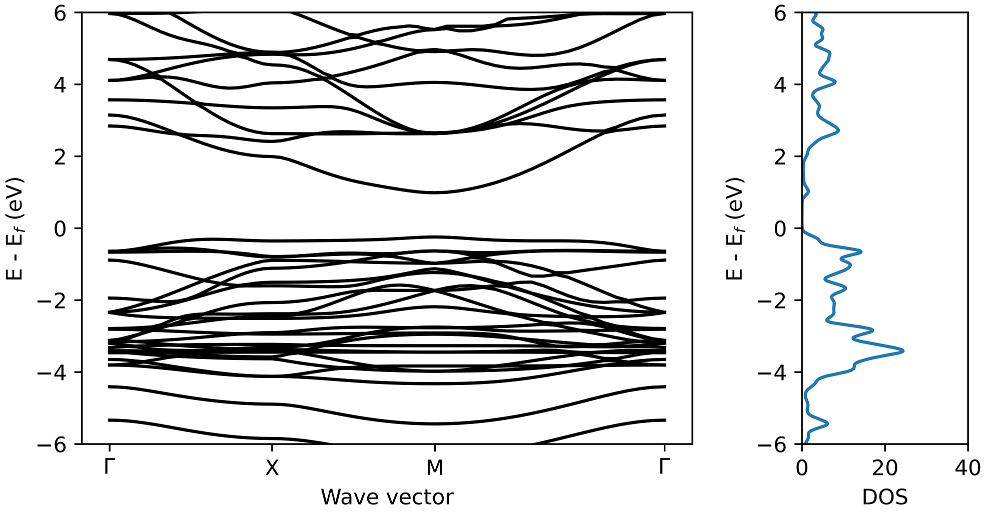

FIG. S11. The electronic band structure and density of states of $AgTlI_2$ calculated using VASP [11] with the PBEsol functional.

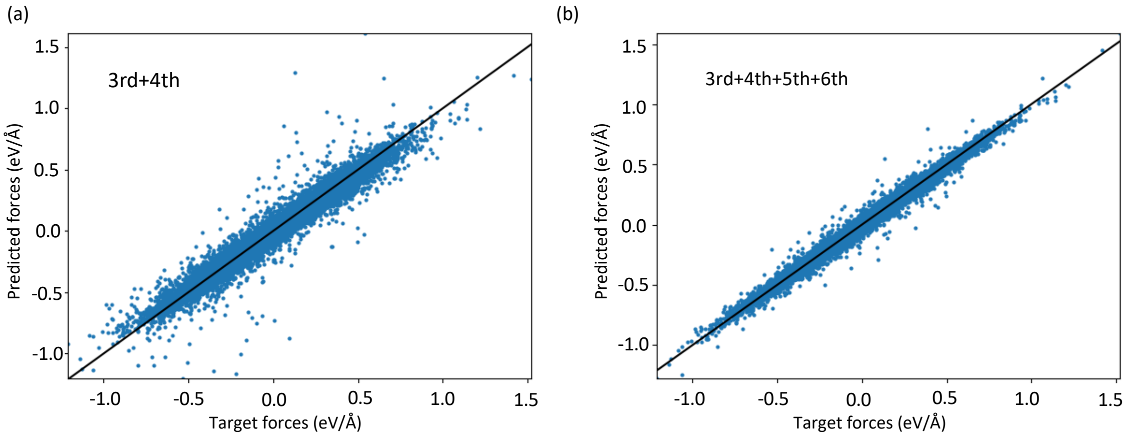

FIG. S12. Fitting performance of the interatomic force constant potential (Taylor expansion) based on the residual force-displacement data. We consider the fitting procedures that include lattice anharmonicity up to the (a) fourth-order and (b) sixth-order terms.

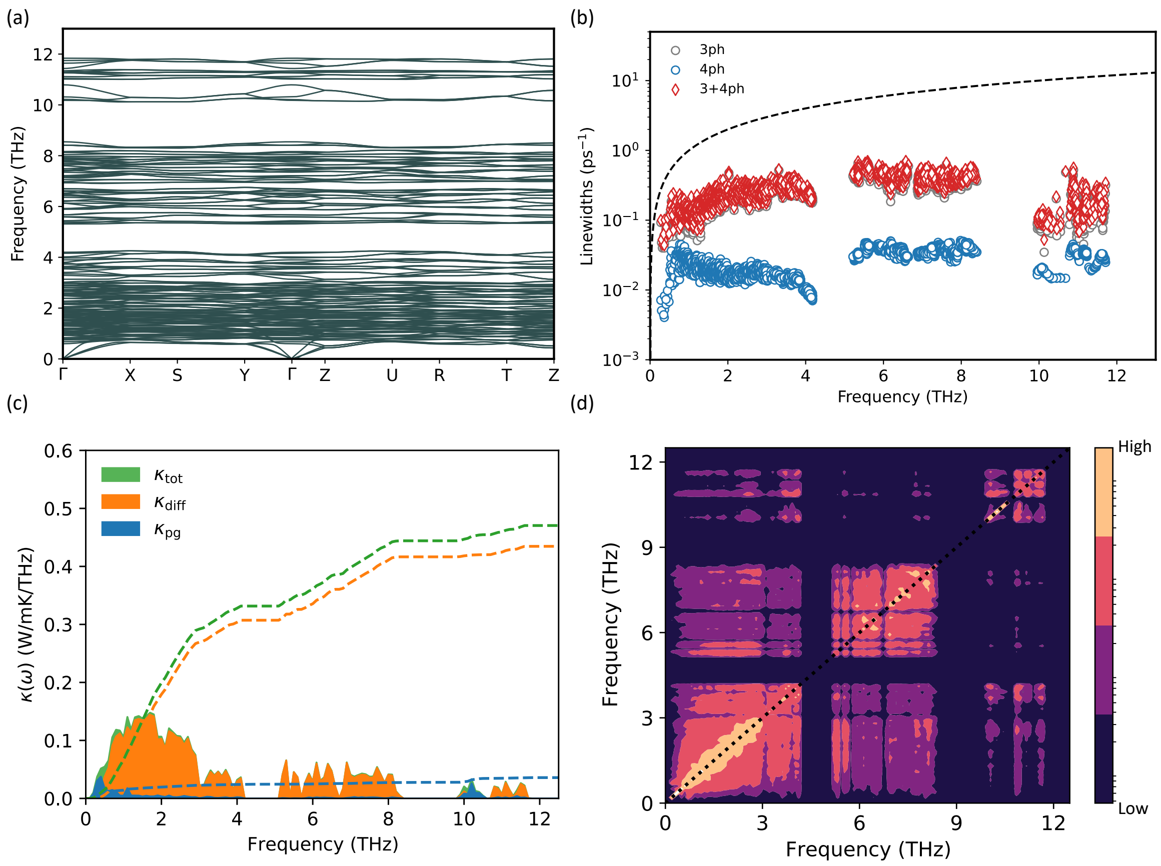

FIG. S13. **Phonon properties and two channel thermal conductivities of Ag_8GeS_6 at 300 K** (a) Phonon dispersion of Ag_8GeS_6 at 300 K calculated using the TDEP scheme. (b) Three- and four-phonon linewidths of Ag_8GeS_6 calculated using the perturbation theory with temperature-dependent atomic force constants at 300 K. (c) Two-channel spectral lattice thermal conductivities and the cumulative values calculated using the unified theory at 300 K, and (d) the two-dimensional frequency-plane distribution.

SUPPLEMENTARY REFERENCES

[1] F. Knoop, *Heat transport in strongly anharmonic solids from first principles* (Humboldt Universitaet zu Berlin (Germany), 2022).

[2] F. Knoop, T. A. Purcell, M. Scheffler, and C. Carbogno, Anharmonicity measure for materials, Physical Review Materials **4**, 083809 (2020).

[3] F. Knoop, T. A. Purcell, M. Scheffler, and C. Carbogno, Anharmonicity in thermal insulators: An analysis from first principles, Physical Review Letters **130**, 236301 (2023).

[4] L. Chen, H. Tran, R. Batra, C. Kim, and R. Ramprasad, Machine learning models for the lattice thermal conductivity prediction of inorganic materials, Computational Materials Science **170**, 109155 (2019).

[5] M. Dutta, S. Matteppanavar, M. V. Prasad, J. Pandey, A. Warankar, P. Mandal, A. Soni, U. V. Waghmare, and K. Biswas, Ultralow thermal conductivity in chain-like tlse due to inherent Tl^+ rattling, Journal of the American Chemical Society **141**, 20293 (2019).

[6] M. Dutta, M. Samanta, T. Ghosh, D. J. Voneshen, and K. Biswas, Evidence of highly anharmonic soft lattice vibrations in a zintl rattler, Angewandte Chemie **133**, 4305 (2021).

[7] K. Pal, Y. Xia, J. He, and C. Wolverton, Intrinsically low lattice thermal conductivity derived from rattler cations in an AMM Q3 family of chalcogenides, Chemistry of Materials **31**, 8734 (2019).

[8] B. Sales, B. Chakoumakos, D. Mandrus, and J. Sharp, Atomic displacement parameters and the lattice thermal conductivity of clathrate-like thermoelectric compounds, Journal of Solid State Chemistry **146**, 528 (1999).

[9] Y. Shi, A. Assoud, C. R. Sankar, and H. Kleinke, $Tl_2Ag_{12}Se_7$: a new pnp conduction switching material with extraordinarily low thermal conductivity, Chemistry of Materials **29**, 9565 (2017).

[10] L. Karanović, D. Poleti, T. Balić-Žunić, E. Makovicky, and I. Gržetić, Two new examples of very short thallium–transition metal contacts: $Tl_3Ag_3Sb_2S_6$ and $Tl_3Ag_3As_2S_6$, Journal of alloys and compounds **457**, 66 (2008).

[11] G. Kresse and J. Furthmüller, Efficient iterative schemes for ab initio total-energy calculations using a plane-wave basis set, Physical Review B **54**, 11169 (1996).
